# The Role of School Adaptation and Self-Concept in Influencing Chinese High School Students’ Growth in Math Achievement

**DOI:** 10.3389/fpsyg.2018.02356

**Published:** 2018-11-29

**Authors:** Danhui Zhang, Yiran Cui, Yuan Zhou, Mengfei Cai, Hongyun Liu

**Affiliations:** ^1^Collaborative Innovation Center of Assessment toward Basic Education Quality, Beijing Normal University, Beijing, China; ^2^Department of Psychology, West Virginia Wesleyan College, Buckhannon, WV, United States; ^3^Faculty of Psychology, Beijing Normal University, Beijing, China

**Keywords:** school adaptation, self-concept, students’ growth percentile, math achievement, social learning theory

## Abstract

A longitudinal designed research study was conducted to provide empirical evidence regarding the influences of three dimensions of students’ school adaptation on their math achievement growth over the first year of high school. These dimensions included learning adaptation, stress management, and personal communication. Student math achievement growth was measured using the student growth percentile (SGP) score. Structural equation modeling (SEM) was used to test for the possible mediating role of self-concept behind those three relationships. Based on the model comparison, it was discovered that school adaptation significantly and positively influences student math achievement growth via mediating effects of student academic self-concept, as opposed to showing a direct impact on students. The findings of this study have important implications for educators and parents to aid students in their pursuit of academic success.

## Introduction

Bronfenbrenner’s (1979) ecological system theory has provided a helpful framework for understanding the importance of adaptation to school. As it explains, each individual interacts with five environmental systems. Among them, the microsystem has the closest bi-directional interactions with students. School, one of the most important microsystems for students, shows profound and enormous influences on their development. Therefore, successful interaction with the school environment and continuous adaptation to school is crucial for all students (Bolger et al., 1998; Margetts, 2009; Huang, 2010, Unpublished). Furthermore, adapting to a new learning stage could be conceptualized as a multifaceted task, which might put considerable pressure on students (Perry and Weinstein, 1998; Valentine et al., 2004). For adolescents, the transition into high school has been identified as a potentially difficult task during which they are faced with many new challenges, including coping with the intellectual, social-emotional, and behavioral demands of a new environment (Estell et al., 2007). A significant number of studies have been conducted to investigate the factors that might influence students’ adaptation capabilities in managing stress and dealing with various difficulties (Dyson and Renk, 2006; Pritchard et al., 2007).

A strong theoretical rationale for the importance of self-concept in students’ school adaptation comes from the perspective of social learning theory. As Bandura (1993) explained, environmental factors are more likely to influence one’s behavior through self-concept, rather than showing a direct effect. Considerable studies have reported that self-concept shows substantial indirect impacts on student engagement and learning outcomes (for example, Arens et al., 2011; Jones et al., 2012). In spite of the abundant investigative approaches exploring self-concept, research regarding its mediating role in school adaptation is, surprisingly, absent. School adaptation could be considered as the process in which students learn how to act in a way suited to a new environment (Bandura, 1977). In such a process, self-reinforcing functions take prominent roles when students set certain performance standards and evaluate their own behaviors. As a result, in this study, self-concept was modeled as an important variable mediating the relationship between school adaptation and later achievement growth.

A literature review discovered important knowledge gaps in the findings on school adaptation. First, most previous research focuses on young children’s adaptation to schools (for example, Ladd et al., 1996; Margetts, 2009; Margetts and Phatudi, 2013; Chi et al., 2018) or college students’ adaptation (Chemers et al., 2001; Lee et al., 2018), while only a few focus on high school students (Ripple and Luthar, 2000; Estell et al., 2007). The challenges of high school life (10th to 12th grade) can be characterized by increased stress, pressure, academic challenges, and higher demands for autonomy. Successful transition and adaptation to high school in the tenth grade might benefit students in multiple ways, such as providing them with a strong academic basis so that they will be prepared for college, as well as enhancing their abilities to monitor and regulate themselves so that they can avoid problematic behaviors. Second, most previous studies analyzed how school adaptation influences students’ outcomes by using cross-sectional data. Few studies have investigated the sustained influences of students’ levels of adaptation on their performance using longitudinal data. Third, despite a considerable number of international studies, Chinese literature on school adaptation is scarce. Few empirical studies have been completed to date exploring the impact of high school adaptation in mainland China. It is worth pointing out that, due to the shortage of higher education resources, the competition for college enrolment is very intense (Kirkpatrick and Zang, 2011; Sellar and Lingard, 2013). Thus, Chinese high school students face higher pressure and anxiety, as well as more academic challenges, compared to their western counterparts at the same stage (Li and Prevatt, 2008; Liu and Lu, 2011; Li et al., 2012; Zhao et al., 2015).

In the present study, longitudinal data based on a sample of Chinese high school students was analyzed to explore the following: (1) how school adaptation at the beginning of tenth grade would influence students’ academic growth throughout the whole school year, while controlling for students’ demographic information; (2) whether students’ academic self-concept serves as a significant variable in mediating the relationship between school adaptation and growth in student math achievement.

## Theoretical Background

### School Adaptation

Adaptation is considered one of the most important capacities of human behavior, and it is also a factor in human motivation and in satisfying human needs (AlZboon, 2013). Regarding school adaptation, earlier existing research focused narrowly on academic achievements. However, Perry and Weinstein (1998) proposed that successfully adapting to the school environment and meeting new expectations and demands is marked by a variety of competencies. As they elaborated, three main competency domains of adaptive performance can be applied to high school students—academic, social, and behavioral. For each domain, different indicators should be considered. In academic competency, students are expected to possess the meta-cognitive skills for learning; in the social domain, students should be capable of building up harmonious relationships with their peers and teachers; and in the behavioral domain, emotional self-regulation is highlighted. In accordance with this explanation and conceptualisation attempts, more and more researchers have explored the construct of adaptation from an “outcome” view (Tsay, 2012). For example, Spencer (1999) regarded school adaptation as a kind of school acculturation with the aim being to maximize the fit between a student’s characteristics and the expectations of the learning environment. Successful adaptation can only be achieved when students respond appropriately to the environment and thus benefit from it (Kaya and Akgün, 2016). Pulakos et al. (2000) also defined adaptation as human beings’ active modification of their own behaviors, so as to adjust themselves to meet the requirements of a new environment. Building on this definition, they constructed the taxonomy of different types of adaptive performance. Adaptation to high school puts higher demands on adolescents to change their own behaviors in three highly related aspects of this taxonomy, including learning adaptability, stress-handling, and interpersonal adaptability.

In this study, we adopted the three domains of functioning in school adaptation, elaborated by Perry and Weinstein (1998), and highlight three important indicators from the taxonomy put forward by Pulakos et al. (2000):

(1) Learning adaptability: For most high school students, their goal is to obtain the knowledge and skills needed to successfully enroll in college; therefore, the capability to acquire the basic knowledge and skills for learning is crucial.
(2) Stress management: After attending high school, students will experience a heightened amount of pressure and a diverse range of difficulties. Therefore, students who can successfully control their frustration, manage their stress, and mitigate their psychological discomfort can facilitate their adjustment to a new environment in a relatively short period of time.
(3) Personal communication: High school students are in the stages of middle and late adolescence; thus, school is one of the most important factors in their social life. The ability to successfully adjust to a new learning environment and to get along with new classmates and teachers will benefit students by providing them with new perspectives.

Previous investigations focusing on the adjustment during the first year of high school have shown that successful adaptation to school has a positive correlation with accomplishment in high school and eventual educational attainment (Ripple and Luthar, 2000; Cadwallader et al., 2002). For example, Ripple and Luthar (2000) investigated a group of ninth grade inner-city adolescents and discovered that better adjustment in school learning and interpersonal relationships predicted a higher high school completion rate. Such a finding was also confirmed in a sample of suburban and non-metropolitan youth in a study conducted by Cadwallader et al. (2002). Estell et al. (2007) examined African-American adolescents from two rural counties and discovered that individuals who experienced a successful transition from middle school to high school had lower rates of substance use and higher grades. Collectively, these studies suggest that students’ improved adaptability to school can be very beneficial in allowing them to maintain positive social relationships, to study efficiently, and to take a positive attitude toward the difficulties and challenges that arise in new learning environments. Eventually, their improved capacity to adjust to new contexts would contribute to better academic performance; for example, they are more likely to engage in their studies (Liu et al., 2014).

### Academic Self-Concept

Shavelson et al. (1976) provided a theoretical definition of self-concept, based on a comprehensive review of existing research, which asserts that an individual’s self-concept is a person’s perceptions of oneself. By proposing a multi-dimensional model with a hierarchical structure, they elaborated that the general self-concept covers different dimensions, including emotional, social, and academic self-concepts. The academic self-concept refers to students’ overall perceptions or beliefs about their academic performance (Shavelson and Bolus, 1982; Marsh and O’Neill, 1984; Marsh, 1990; Wigfield and Karpathian, 1991; Byrne and Gavin, 1996). The academic self-concept should also be considered subject-specific. For example, students’ strong self-concept in math does not necessarily transfer to other subjects, such as reading (Marsh, 1986; Marsh and Yeung, 1997; Arens et al., 2011; Jansen et al., 2015). Moreover, it was also discovered that if the subject of academic self-concept and achievement were matched, the effect of such a relationship is much higher than when only a general self-concept was considered (Jansen et al., 2015).

As explained in the self-enhancement model, self-concept is a determinant variable of academic achievement (Calsyn and Kenny, 1977). Students with higher academic self-concept are confident in learning, with stronger motivation and more persistent engagement (Nagengast and Marsh, 2011). Considerable empirical evidence supports this significantly positive and direct effect of academic self-concept on students’ academic performance (Calsyn and Kenny, 1977; Shavelson and Bolus, 1982; Marsh, 1990; Hamachek, 1995; Fenollar et al., 2007; Marsh and Martin, 2011; Green et al., 2012). This relationship trend was also clearly identified in a meta-analysis review conducted by Huang (2011).

A student’s math-related self-concept refers to the perceptions or beliefs of his or her ability to do well in math (Shavelson and Bolus, 1982). Many empirical studies using cross-cultural data from large-scale international assessments have revealed moderate-size effects of the relationship between math self-concept and math performance (Marsh and Hau, 2004; Wilkins, 2004; Marsh et al., 2006; Chiu, 2008; Shen and Tam, 2008; Yoshino, 2012). For example, Wilkins (2004) studied the self-concept of students who participated in the Trends in International Mathematics and Science Study (TIMSS) 1995 and investigated the relationship between students’ self-concept and their achievement in both math and science at the individual and country level. However, only the relationship with math achievement was statistically significant and positive at the individual level. This result is consistent with what was found in Chiu’s (2008) research using data from the TIMSS 2003. In addition, by employing the TIMSS 2007 assessment results, Yoshino (2012) conducted a cross-cultural comparative study of the influences of self-concept on academic math achievement. It was discovered that in both the United States and Japan, math self-concept shows significantly positive impacts on students’ academic performance; nevertheless, the strength of such a relationship varies in the two countries. Similar findings indicating that self-concept can significantly predict student academic performance were revealed in other studies based on data from the Programme for International Student Assessment (PISA) (Marsh and Hau, 2004; Marsh et al., 2006). For example, the analysis of PISA data from 25 countries suggests positive cross-cultural invariant association between these two variables, with an average correlation coefficient of 0.33 (Marsh et al., 2006).

### Theoretical Framework From the Perspective of Social Learning Theory

Past research suggests an empirical relationship between academic self-concept and achievement. In addition to the investigations of the direct relationship between these two variables, research has been focused on the mediating role of self-concept in the relationship between other factors and learning outcomes (for example, Arens et al., 2011; Jones et al., 2012). What is the theoretical rationale behind this exploration? As Bandura (1993, 1997) explained, environmental factors are more likely to influence one’s behavior through self-concept, rather than showing a direct effect. In the framework of social learning theory, self-concept, serving as a function of self-reinforcement, signifies persons’ tendencies to self-approve or self-criticize their own behaviors (Bandura, 1977). Such a control mechanism of self-concept is very important since it enables people to control their own behaviors more effectively. For example, students’ perceptions of their friends’ behaviors could influence their math self-concept, which in turn influences their math performance (Jones et al., 2012).

From the perspective of social learning theory, self-concept could act as an important vehicle for addressing the difficulties in school adaptation. When students engage a new environment, they start by learning from external standards to understand what actions are appropriate in this new context. How do they regulate their adaptation behaviors? They receive some feedback from others, such as teachers or peers. However, students’ behaviors are not determined solely by external information. Self-concept takes a more important role in regulating students’ behaviors, whereby students will compare their own behaviors with the perceived standards and make self-evaluations. In this way, students’ self-concept can facilitate their progress toward acquiring the knowledge and skills necessary for the successful transition into a new learning environment. Furthermore, their self-concept will influence their academic achievement in the long run.

### The Present Study

In the current study, we explored whether self-concept also serves as a mediating variable affecting the relationship between school adaptation and academic performance. We particularly focused on the sample of Chinese high school students, with the hope that this study will provide insights for researchers from other cultural contexts. In China, high school education is not part of the compulsory education curriculum. Students are ranked and selected based on their performance on the High School Entrance Test for high school enrolment. Even if they are successfully enrolled, adapting to this new environment is potentially a difficult task in which they are faced with three main challenges. First, compared with middle school (7th to 9th grade), the difficulty level of the learning content and the curriculum standards are raised substantially. Second, high school teachers tend to put students under the pressure of college entrance from the very beginning. If students are not able to quickly adapt to their new learning environment, they might be left behind in the long run. Third, students experience pressure and competition from their peers. Many students were at the top of their class in middle school; however, they must accept the fact that this may no longer be the case.

In this study, student self-concept in learning was modeled to mediate the relationship between school adaptation and student achievement in math. More specifically, it was hypothesized that students who possess better school adaptation levels will have higher self-concept, and thus their achievements in math will grow faster. The main research questions included: (1) whether students’ adaptation in the beginning of the semester in high school will influence their math achievement gain at the end of the first school year; and (2) whether students’ academic self-concept in math will mediate the relationship between these two variables.

## Methodology

### Sample

This study analyzed secondary data from a large-scale education assessment collected in Zhengzhou, the capital city of Henan province in China. All schools in Zhengzhou agreed to participate in the assessment, which was reviewed and approved by the research committee of Beijing Normal University, as well as by the local government committee. The school teachers, students, and their parents had a clear understanding of this project and how data was collected. The parents of all student participants approved and signed informed consent forms. In all, 10,487 students from 45 high schools were sampled; 40.4% of them were female and 58.9% were male (0.7% did not respond on this question). A longitudinal study was designed to address the influence of school adaptation throughout an entire 10th grade school year. At the beginning of the first semester of 10th grade, all of the student participants took a survey which collected and evaluated their demographic information, their school adaptation capability, and their self-concept in math learning. Math achievement tests were also administered at the beginning and the end of their 10th grade year. To protect individual privacy, all participants were anonymous in this study.

### Measures

Demographic information included student gender and social economic status (SES), which was measured by assessing the following variables: father’s highest education level, mother’s highest education level, father’s vocation, and mother’s vocation. The final SES score constituted an integrated index of the above four variables based on the principle component analysis.

*Student school adaptation* consisted of three components: learning adaptation (six items), stress management (three items), and personal communication (four items). Responses were rated on a 5-point Likert scale from 1 (definitely did not meet) to 5 (definitely did meet). The sample items for respective dimension included: “I know how to handle the new learning task in high school” (learning adaptation); “I can control my stress in the new high school” (stress management); “It is easy for me to make new friends” (Personal communication). Cronbach’s alpha indices for each dimension were 0.85, 0.75, and 0.78, respectively. According to the confirmatory factor analysis (CFA), all the item factor loading exceeded 0.4, both the CFI and TLI were above 0.95, and the RMSEA fell below 0.08, which indicates high construct validity (see Table 1 for detailed information).

**Table 1:** Confirmatory factor analysis on latent constructs.

Dimension	χ^2^	*df*	CFI	TLI	RMSEA	Loading range
Learning adaptation	70.8	9	0.95	0.93	0.07	0.41–0.77
Stress management	0	0	1	1	0	0.44–0.62
Personal communication	57.1	2	0.99	0.97	0.05	0.52–0.67
Math Self-concept	1387.1	14	0.95	0.93	0.07	0.59–0.83

*Student academic self-concept* in math was the core variable that served as the mediating variable in the hypothesized model. Similar to school adaptation, it was measured using a Likert scale with seven items, assessing students’ perceptions about whether they were able to succeed in learning. The sample items included: “I usually could achieve the goal in learning math,” and “I think I perform pretty well on math.” The Cronbach’s alpha (0.87) and model fit index (CFI = 0.95, TLI = 0.93, RMSEA = 0.08) from the CFA indicate high reliability and validity.

*Student math achievement growth* was measured according to the student growth percentile (SGP) score, which examined students’ current achievement relative to other students who were at the same position in the beginning (Slaughter, 2008; Betebenner, 2011). In other words, SGP measures student progress by comparing one student’s progress with the progress of the peers who had similar scores in their prior performance. SGP score has been used in large-scale assessments to measure individual student progress; for example, the state-wide assessment Massachusetts Comprehensive Assessment System (MCAS) conducted in the United States used SGP to track student scores from one year to the next.

To quantify a student’s SGP score, the first step is the estimation of the conditional density of a student’s current score, while taking this student’s prior score as the conditioning variable. Second, given the conditional density obtained at step 1, the student’s SGP is defined as the percentile of the score within that conditional density (Briggs and Betebenner, 2009). The calculation of conditional density is based on a quantile regression model (Koenker, 2005), where 100 was the maximum possible score, and in which 1 corresponded to each percentile. In the current study, the curve-linear functional relationships between a student’s current score (end of 10th grade) and previous score (beginning of 10th grade) were assessed. The cut score for each percentile corresponding to the dependent variable was calculated using the combinations of the regression coefficients and prior scores. The percentile cut score scale demonstrates the growth percentile for each student based on his or her real score at the end of 10th grade and indicates the progress toward achievement (Betebenner, 2011).

Student growth percentiles range from 1 to 99, where higher numbers represent higher growth and lower numbers represent lower growth. With SGP scores, students who had made remarkable progress would stand out from those who had not. Given that the SGP score measures relative performance rather than absolute performance, how students performed in their previous test is irrelevant (Chester, 2011). In the study, each sampled student received a SGP score, which measures, over their 10th grade school year, how much the student changed in math achievement relative to other students with similar scores in the beginning. For example, if a student has a SGP score of 95 at the end of 10th grade, it means that this student grew as much as or more than 95 percent of his or her peers who had similar scores at the beginning of 10th grade. Calculation of students’ SGP scores was conducted in R software using the SGP Package (Betebenner and Iwaarden, 2011).

### Data Analysis

The data analysis consisted of two parts: A descriptive analysis was conducted to provide basic descriptive statistics for each variable. Then, a structural equation modelling (SEM) analysis was used to investigate the research questions regarding how different dimensions of school adaptation influenced student achievement growth, and whether or not academic self-concept demonstrated mediating effects with those relationships. We first tested the hypothesized model where all three components of school adaptation influence the student achievement growth in math directly, and then we examined the second hypothesized model where students’ academic self-concept in math mediates the relationship between three components of school adaptation and achievement gain. Through comparing these two models, evidence supporting the mediation effect of self-concept could be collected.

A SEM analysis was selected as an analysis tool due to its ability to deal with the complex relationships among different variables and to control for measurement errors. The overall model fit was evaluated based on the following index: if the Root Mean Square Error of Approximation (RMSEA) was below 0.08, and if the Tucker-Lewis Index (TLI) and Comparative Fit Index (CFI) were greater than 0.90, this was considered a satisfactory fit (Schreiber et al., 2006). In addition, if all of the loading values of the latent factor items exceeded 0.4, this was also considered a satisfactory fit. The path coefficient in the model was tested using T statistics to determine whether the hypothesized relationship was significant. In order to understand how school adaptation affected math achievement gain in a general sense, we calculated the effect size of the indirect effects, which equals the product of the two path coefficients in the mediating relationship. The mediating effect of self-concept was also examined by a Sobel test (Sobel, 1982). The entire analysis was conducted using Mplus 7.0 Software.

## Results

### Hypothesized Model 1

Did the school adaptation at the beginning of 10th grade influence students’ academic growth over the course of one school year directly, while controlling for students’ SES and gender?

**FIGURE 1:**
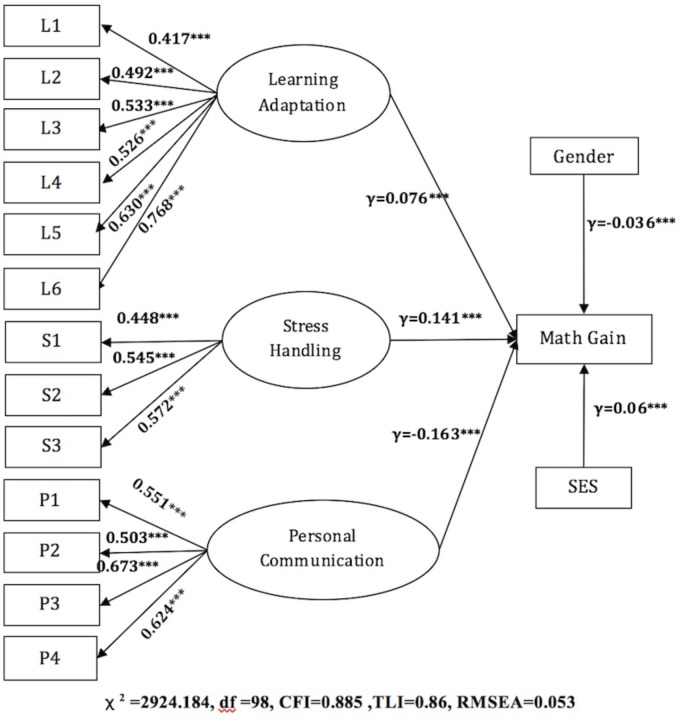
The influences of school adaptation on student math growth. The loadings in the SEM figures: ^∗∗∗^ indicates the pathway was significant at 0.001 or below (*p* < 0.001).

All three components of school adaptation (learning adaptation, stress management strategy, and personal communication skills) and student demographic information were examined (see Table 2 for descriptive statistics). Regarding their direct relationship with student growth in math achievement (Figure 1), it was found that while controlling for student socio-economic status and gender, the three components of school adaptation showed mixed effects on student achievement growth. A closer look at the three components revealed that both learning adaptation (γ = 0.076, *p* < 0.000) and stress-management skills (γ = 0.141, *p* < 0.000) had significant and positive effects on math achievement growth. However, this trend was not observed in personal communication skills. Contrary to the hypothesis, there was a significantly negative relationship between student personal communication skills and student growth in math achievement (γ = -0.163, *p* < 0.000).

**Table 2:** Descriptive statistics and correlations among the variables.

Construct	*N*	Mean	*SD*	2	3	4
1. Learning Adaptation	10495	3.35	0.76	0.217^∗∗^	0.207^∗∗^	0.556^∗∗^
2. Stress Handling	10495	3.16	0.87		0.422^∗∗^	0.281^∗∗^
3. Personal Communication	10495	3.47	0.98			0.268^∗∗^
4. Self-concept	10495	3.52	0.75			

*Note: ^∗^indicates the correlation was significant at 0.05 or below (*p* < 0.05), ^∗∗^indicates the correlation was significant at 0.01 or below (*p* < 0.01)*.

The positive influence of SES on student achievement gains (γ = 0.06, *p* < 0.000) was confirmed by the model, which aligned with our hypothesis. Students from the higher SES families showed faster growth in math achievement. However, female students were revealed to grow slower in math compared with their male peers (γ = -0.036, *p* < 0.000) (see Figure 1). All the items had a high correlation with the latent factors, ranging from 0.417 to 0.768.

However, despite that all the paths are significant, the overall model fit indices demonstrated that the hypothesized model does not fit the observed data very well. The overall model fit was poor, with low CFI and TLI indices (CFI = 0.885, TLI = 0.860). According to this outcome, it is hard to find evidence of the direct influence between learning adaptation, personal communication, and stress management with achievement growth in math.

### Research Question 2

Did students’ academic self-concept in math serve as a significant variable in mediating the relationship between school adaptation and student achievement growth?

**FIGURE 2:**
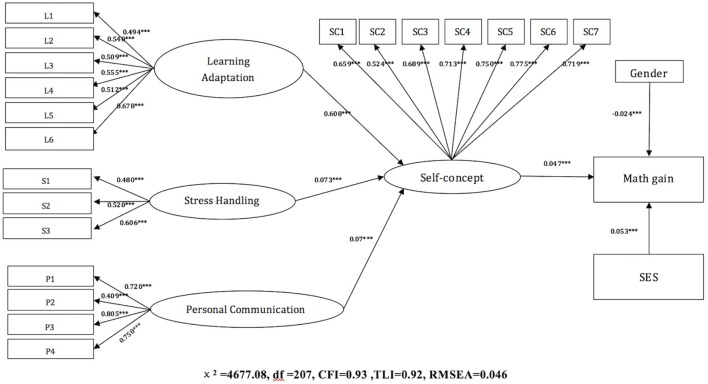
The SEM model with the mediating effects of self-concept for all three dimensions. The loadings in the SEM figures: ^∗∗∗^indicates the pathway was significant at 0.001 or below (*p* < 0.001).

With the aim to answer the second research question, the second SEM model was established to analyse the mediating effect of academic self-concept. Student learning self-concept was used as a mediating variable that connected these three constructs with student achievement growth, while gender and SES were controlled as the confounding variable. While integrating all three components into one model, it is also possible to explore the relative strength of the mediating links between three components of school adaptations and self-concept, as well as the relationship between self-concept and growth in math achievement (see Figure 2). It was discovered that all three components of school adaptation (learning adaptation, stress management, and personal communication) demonstrated a positive influence on student self-concept in learning, which in turn was positively related with student math gains.

First, according to the SEM analysis results, learning self-concept was a significant mediating variable in forming the relationship between learning adaptation and achievement growth when both the student gender and SES variables were controlled. Both the path coefficient from learning adaptation to self-concept (γ = 0.608, *p* < 0.000) and the one from self-concept to math SGP score (γ = 0.047, *p* < 0.000) were positive and significant. This indicated that if students have higher learning adaptation, they are more likely to have higher self-concept, and thus they will achieve faster academic growth. The mediation effect in this model was calculated by multiplying these two path coefficients, which resulted in 0.029 (*p* < 0.000). Given that this mediation relationship was discovered to be significant in a Sobel test, we were able to conclude that the overall mediation effect was significant (Table 3).

**Table 3:** Mediating effect of school adaptation on math growth via self-concept.

	Indirect effect	*SE*
Learning adaptation to self-concept to math growth	0.029^∗∗∗^	0.007
Stress management to self-concept to math growth	0.003^∗∗∗^	0.001
Personal communication to self-concept to math growth	0.003^∗∗∗^	0.001

*Note: ^∗∗∗^indicates the effect was significant at 0.001 or below (*p* < 0.001)*.

The relationships among stress management, self-concept, and academic growth were quite similar compared to the previous pattern. The academic self-concept was found to have a significant and positive mediating role on the association between stress management and math SGP scores. The path between stress management and learning self-concept was significantly positive (γ = 0.073, *p* < 0.000), as well as the path between self-concept and growth in math achievement (γ = 0.047, *p* < 0.000). Therefore, students who have stronger skills in managing stress tend to possess higher self-concept, and thus their achievement in math tends to grow faster compared with their peers. The indirect mediating effect in this model was significant with the value of 0.003 (see Table 3).

The relationships among students’ personal communication skills, learning self-concept, and student gains in math also showed a similar pattern. The significant mediating role of self-concept with relation to student personal communication and math gains was observed. Students’ personal communication skills positively influenced students’ learning self-concept (γ = 0.07, *p* < 0.000), which in turn positively affected their math gain (γ = 0.047, *p* < 0.000). Thus, the overall mediation effect was found to be positive with the Sobel test (γ = 0.003, *p* < 0.000) (see Table 3).

In the second SEM model, SES and gender demonstrated significant influences on achievement growth. In line with our predictions, the relationship between SES and growth in math achievement was significant and positive, while the relationship between gender and the outcome variable was negative. It can be concluded that higher SES correlates with higher academic growth, while female students experienced slower growth compared with their male counterparts.

Comparing with the first model, the overall model fit of the second model was much better and achieved a good to excellent model fit of the real data. The CFI and TLI indices were 0.93 and 0.92, respectively, and the RMSEA was 0.046. All the factor loadings for the items of each latent variable exceeded 0.4. As a result, the second model indicated a stable and robust mediating relationship of academic self-concept. According to this result, students who were able to better handle their stress, to adjust their learning strategies and behaviors, and to build up harmonious relationships with teachers and peers, were more likely to establish a positive and stronger self-concept, and thus achieve higher growth in math achievement. However, among all three components, learning adaptation showed the strongest influences.

## Discussion and Significance

This research focused on 10th grade Chinese high school students and investigated their experience in the first year of their high school lives. The most important reason why we pay attention to this group of students is that adolescents who have just transferred from middle school to high school are faced with multiple kinds of new challenges they have never before encountered. In the current study, school adaptation is defined from the point of view of “outcome,” which is a student’s capacity to adapt to a new learning environment so that they may achieve success and find fulfillment. Three sub-domains of school adaptation were identified: learning adaptation, stress management, and personal social communication. Through longitudinal design, we examined students’ growth in math achievement and how this growth was influenced by various factors related to their learning experience.

One of the most important contributions of this study is to inform the literature on school adaptation by providing empirical evidence supporting the assertion that school adaptation can positively influence student achievement growth based on longitudinal data. By analysing the growth in percentile scores, students’ improvements in math achievement over the first year of high school were quantified. Such a finding is important since few of the previous studies in this field employed longitudinal data to measure gains in student achievement.

The second contribution of the current study concerns our examination of the psychological mechanism behind the relationship between school adaptation, self-concept, and achievement gains in math. Self-concept, defined as one’s general perception about oneself (Bong and Clark, 1999), has been considered a core factor that has historically influenced students’ general performance. According to the social learning theory, self-concept strongly relates to how one behaves, especially when faced with difficulties and challenges (Bandura, 1977). In the current study, self-concept was not only highlighted by its direct relationship with academic performance, but also by its indirect effects in predicting future academic performance. Regarding its direct effects, the observed positive and strong relationship between self-concept and academic achievement confirmed the previous conclusion drawn from a comprehensive review of 143 articles (Ma and Kishor, 1997). In terms of the indirect effects of self-concept, the finding about its mediating effects contributes to the existing literature discussing the influential mediating role that self-concept plays in connection to many diverse psychological attributes and academic achievement (Arens et al., 2011; Jones et al., 2012).

### The Relationships Between Learning Adaptation and Growth in Math Achievement via the Critical Mediating Effects of Self-Concept

Consistent with our hypothesis, students’ learning adaptation had a positive relationship to student academic growth, via the mediating effect of academic self-concept. Successful learning adaptation mainly indicated that students could acquire sufficient knowledge and skills to meet basic academic requirements. The findings of the positive relationship between learning adaptation and academic gains demonstrated that students who have a higher capacity to adjust to a new learning environment in the beginning of a semester are more likely to achieve faster improvement in math during the semester. Moreover, if students’ self-concept is considered, the reason why learning adaptation positively influences achievement becomes even clearer. It is because students who were able to adapt to a new learning environment at the beginning of high school (10th grade) had more confidence in themselves in terms of completing learning tasks; thus, they were more likely to have higher expectations of themselves, and they tended to improve in math faster through the whole 10th grade. It is also worthy of note that learning adaptation showed the strongest impact on self-concept out of all three factors. The primary reason for this is the fact that learning adaptation is the one indicator that has the closest relationship with learning self-concept.

### The Relationships Between Stress Management and Growth in Math Achievement via the Critical Mediating Effects of Self-Concept

According to previous findings (Andrews and Wilding, 2004; Dyson and Renk, 2006), students’ stress levels have a negative direct influence on their academic performance. In this study, stress-management skill, defined as the capability to handle stress, was discovered to positively affect student math gains through the mediating variable of self-concept. Students face a greater amount of diverse challenges after entering high school and tend to experience increasing pressure. It is common for some students to suffer from frustration, anxiety, and loneliness. All of these negative feelings tend to affect students as they adapt to their new school life, and will consequently influence their academic performance. Therefore, students who are able to mitigate their psychological discomfort efficiently tend to have better perceptions about themselves when it comes to learning math, so they achieve faster growth in math compared to their peers who are unable to do so.

### The Relationships Between Personal Communication and Growth in Math Achievement via the Critical Mediating Effects of Self-Concept

Consistent to our hypothesis, after controlling for student gender and socio-economic status, the relationship between student personal communication and student performance growth as mediated by the effect of self-concept was significantly positive—which indicated that students who reported higher personal communication skills would acquire stronger self-concept, and in turn have higher academic growth. Such results are actually consistent with our common sense, which is that harmonious social relationships can influence students’ self-evaluation and can ultimately influence their academic achievements. As some previous researchers have pointed out, establishing friendships with new classmates and getting along well with teachers might benefit students by providing them with different perspectives extending beyond academic achievements. What was revealed in this study provides additional evidence showing that personal communication can have a positive and significant influence on student self-concept, which furthermore affected students’ improvement in math.

### Key Implications for Educators and School Practices

Difficulties in adapting to school might lead to further problems for students. Existing studies have suggested that people’s behavior and psychosocial problems in adulthood can be traced back to their failure in early school adaptation (Bolger et al., 1998; Margetts, 2009; Huang, 2010, Unpublished). Therefore, increasing understanding of adolescents’ adaptation at schools will contribute to establishing interventions that are responsive to students’ diverse needs in adaptation in real educational practice.

The implications of this research suggest two important ways in which we can support high school students. First of all, as social learning theory (Bandura, 1977) states, human learning is a cognitive process which involves modeling. One important type of modeling stimuli addressed by Bandura is “verbal instruction.” Participants could learn from the detailed instruction or guidance of others. Therefore, if we could provide sufficient guidance for newly enrolled high school students, they could receive more support and, in turn, be able to adjust their learning strategies to successfully achieve their learning objectives. For example, teachers could describe the desired learning behaviors in detail, including how to review, question, think critically, or learn independently, and then instruct students to engage in these behaviors. Teachers can also establish peer or team support groups to encourage students to help and learn from each other.

Second, according to social support theory (Cohen and Wills, 1985), strong social support is considered a useful coping resource for people as they experience life changes. A large number of studies have indicated that social support can buffer negative psychological feelings (Boulter, 2002; Tsay, 2012), especially students’ stress levels (Calicchia and Graham, 2006). School is an important component of social life for high school students, which plays a significant role in determining their social functioning capacity (Chen et al., 2011). Educators should pay close attention to students’ psychological health and offer high-quality instructions for mitigating their stress, which is essential for students who are in the critical stage of high school as they might lack the capacity to find outlets for pressure and stress. Particularly, school administrators are encouraged to hold workshops for stress management (Pritchard et al., 2007) to help those students with severely high stress levels. These students may be regarded as “selfish” or “weird,” or they are more likely to be rejected by their peers and perform poorly in school, and thus have low self-concept (Chen et al., 2011). The psychological support from school can strengthen their willingness to be positive and optimistic, and eliminate their emotional arousal, so as to improve their self-concept. Another way to provide strong social support is to encourage students to organize different activities, student events, or student clubs, and encourage students to participate in them. In this way, students will establish strong friend circles for themselves at school, which will increase the likelihood of developing a high self-concept.

This study provided insights about the effects of school adaptation and contributes to the exiting literature in multiple ways. However, the limitations should also be pointed out. First, although the longitudinal data might provide insights and tentative evidence for a possible causal relationship, precautionary measures should be taken to not draw concrete conclusions from causal inferences. Second, only academic self-concept was addressed in this study as a mediating variable. Similarly, only the subject of math was explored as the learning outcome. It is worthwhile to explore other possible factors that might demonstrate potential mediating influences on the relationship between school adaptation and growth in other subjects.

## Author Contributions

DZ and HL made significant contribution to the conception, research design, and drafting. YC and YZ conducted the data analysis and finalize the document. MC contributed to the interpretation of the data and revising the document critically.

## Conflict of Interest Statement

The authors declare that the research was conducted in the absence of any commercial or financial relationships that could be construed as a potential conflict of interest.
